# The Use of Smartphone Serious Gaming Apps in the Treatment of Substance Use Disorders: Observational Study on Feasibility and Acceptability

**DOI:** 10.2196/34159

**Published:** 2022-09-06

**Authors:** Thelma Schilt, Elvira Sharine Ruijter, Nikky Godeschalk, Marit van Haaster, Anna E Goudriaan

**Affiliations:** 1 Jellinek, Amsterdam Institute for Addiction Research Arkin Mental Health Care Amsterdam Netherlands; 2 Department of Psychology, Brain&Cognition University of Amsterdam Amsterdam Netherlands; 3 Department of Psychiatry Amsterdam UMC, University of Amsterdam Amsterdam Netherlands; 4 VU-University Medical Center, School of Medical Sciences Vrije Universiteit Amsterdam Amsterdam Netherlands; 5 Department of Research and Quality of Care Arkin Mental Health Care Amsterdam Netherlands

**Keywords:** addiction care, mobile phone, cognitive training, neurocognition, mental health, mobile health, digital applications, health applications, smartphone, cognitive assessment

## Abstract

**Background:**

Addiction is a worldwide problem with major health complications. Despite intensive treatment, relapse rates remain high. The prevalence of cognitive impairment is high in patients with substance use disorders (SUDs) and is associated with treatment dropout and relapse. Evidence indicates that cognitive function training in persons with SUDs may support treatment. Therefore, the use of web-based tools to test and train cognitive functions is of increasing interest.

**Objective:**

The goal of this study was to determine the feasibility and acceptability of a serious gaming smartphone app to test and train cognitive functions in addition to the treatment of SUDs.

**Methods:**

A prospective observational study was conducted with 229 patients seeking addiction treatment. The patients were offered 2 smartphone apps in addition to regular care: MyCognition Quotient (MyCQ) assessed cognitive functions and AquaSnap trained these functions. The feasibility was determined based on acceptance rates. The acceptability of the smartphone apps was qualitatively analyzed based on the answers to a questionnaire. Patient characteristics were compared between patients who played and did not play smartphone games. Explorative correlation analyses were performed between the playing time and cognitive assessment scores.

**Results:**

Of the 229 patients who were offered the apps, 110 completed the MyCQ assessment, and 59 started playing AquaSnap, yielding acceptance rates of 48.0% and 25.8%, respectively. The group that completed the MyCQ assessment was significantly more educated than the group that did not download the apps (*χ*^2^_2_=7.3; *P*=.03). The education level did not differ significantly between the group that played AquaSnap and the group that did not (*P*=.06). There were relatively more women in the AquaSnap playing group than in the nonplaying group (χ^2^_1_=6.5; *P*=.01). The groups did not differ in terms of age, substance use, treatment setting, mood, or quality of life. With respect to acceptability, 83% (38/46) of the patients who filled out the questionnaire enjoyed taking the MyCQ measurement, whereas 41% (14/34) enjoyed playing the AquaSnap game. Furthermore, 76% (35/46) and 68% (23/34) rated the apps MyCQ and AquaSnap, respectively, as easy. More playing minutes was associated with decreased working memory reaction time and executive functioning accuracy.

**Conclusions:**

Our study showed that the use of a smartphone app for cognitive assessment in patients with SUDs who are interested and highly educated is feasible and acceptable for the subgroup that was asked to fill out a perception questionnaire. However, the use of a smartphone app for cognitive training was less feasible for this group of patients. Improvement of the training application and enhancement of the motivation of clients are needed. Despite these limitations, the present results provide support for future research investigating the use of smartphone apps for cognitive assessment and training in relation to the treatment of SUDs.

## Introduction

### Background

Worldwide, approximately 269 million people used drugs in 2018 (World Drug Report 2020, United Nations Office on Drugs and Crime), and among them, an estimated 35.6 million people had substance use disorders (SUDs). Approximately 12.5%, that is, 4.5 million people with SUDs received treatment. A common intervention is psychological therapy, such as cognitive behavioral therapy, motivational interviewing, and contingency management [1,2], often in combination with medications. Although there is evidence that these interventions are effective [1,2], relapse rates remain high [3], and approximately half of the patients treated for SUDs relapse within 1 year after treatment [4]. Dropout is a predictor of relapse [5], with nearly one-third of the patients dropping out of psychosocial SUDs treatment [6]. One of the risk factors for dropout from treatment is the presence of cognitive deficits [7]. Moreover, cognitive impairment is associated with an increased chance of addiction relapse [8,9], even after recovery from SUDs [10]. Bruijnen et al [11] showed that cognitive impairment was present in 31% of patients with SUDs who presented for treatment. Substance use can impair executive functioning, such as decision-making, flexibility, planning, and inhibition [12], and in addition, weaker executive functions have been related to the development of addictive disorders (eg, [13,14]). Depending on the substance abused, specific cognitive functions may be affected [12,15,16]. Moreover, different cognitive impairments predict relapse, and training could be aimed at improving those cognitive functions [10].

Early detection of cognitive impairment in addiction care is of clinical importance, because cognitive training can be provided in time to optimize addiction treatment. A combination of behavioral therapy and cognitive training may improve treatment outcomes [17]. Preliminary evidence shows promising results for cognitive training in addiction treatment [18]. A recent review article supported the usefulness of structured cognitive training programs in addition to conventional addiction treatment to improve cognitive performance in patients with SUDs; however, a limited number of studies have also evaluated SUD clinical outcomes, such as substance reduction or relapse prevention [9]. Improved cognitive functioning can lead to better social inclusion and support [19].

The content, methods, and applications of cognitive assessments vary widely, and the requirement for specialist supervision is time consuming and expensive. In the search for cheaper and more efficient methods, mobile apps are of increasing interest. Mobile apps have good accessibility and low costs and can be used to monitor and potentially improve mental health [20]. Previous research in adolescents who abused alcohol showed a significant improvement in behavioral control in adolescents who were trained with a serious game based on a stop-signal paradigm [21]. In addition, improvement in frontal cognitive functioning after training with a serious game was found in patients undergoing alcohol rehabilitation [22]. Another study in male Veterans with alcohol use disorder suggests that serious games that emphasize relapse prevention intervention techniques have positive effects on self-reported ratings of alcohol dependence, alcohol craving, and self-efficacy [23]. In a study of patients dependent on heroin, undergoing methadone maintenance treatment, a serious game was used in the treatment program, but because of the small sample size, it was uncertain whether the improvement at follow-up was due to the cognitive intervention [24].

### Objectives

A web-based tool that can be run on a smartphone, MyCognition Quotient (MyCQ), was developed to quickly and easily assess the broad cognitive status of patients. The app was validated in patients with obsessive-compulsive disorder, schizophrenia, and major depressive disorder [25]. MyCQ is used in unison with a web-based training application AquaSnap, where it tracks progress and determines which cognitive domains require the most training. Recently, the beneficial effects of the training application AquaSnap on the perception of subjective cognitive functioning were found in a group of patients with breast cancer [26]. It is of great value to investigate whether these 2 applications are useful in patients with SUDs. As advised by the National Institute of Health Research, this study focused on the feasibility of such a study [27]. As these smartphone apps have not yet been studied for the treatment of addiction, their compliance and acceptability are not known. Therefore, this study examined the feasibility and acceptability of these apps in a group of patients with SUDs in an addiction treatment setting. The association between playing time and cognitive functioning was explored in patients who used the cognitive training application.

## Methods

### Ethics Approval

This study was categorized as not subject to the Medical Research Involving Humans Subjects Act (Wet Medisch-wetenschappelijk Onderzoek met mensen) by the Medical Ethics Committee of the Amsterdam University Medical Centers. Informed consent was obtained from all participants before the start of the study, and the study was performed in accordance with the Declaration of Helsinki.

### Study Settings and Design

In a prospective study, the feasibility of implementing serious games in addition to addiction care was investigated. The target population was patients seeking treatment in an addiction-treatment center, Jellinek, Amsterdam, comprising an outpatient and inpatient facility, including a detoxification facility. Treatment was voluntary, and the patients and participants could stop treatment at any time. Convenience sampling was used to recruit patients.

### Participants

Participants were recruited between April 2019 and June 2020. Patients starting cognitive behavioral therapy or acceptance and commitment therapy at the detoxification, inpatient, or outpatient units were informed regarding the study through leaflets and presentations. Interested patients could participate in the study if they fulfilled the criteria of substance use or gambling disorder according to the Diagnostic and Statistical Manual of Mental Disorders—Fifth Edition, were aged between 18 and 75 years, could read Dutch or English, and were capable of using a smartphone. Patients were excluded if they were addicted to gaming or gambling on their smartphones or the internet.

### Measures

#### Patient Characteristics

Demographic and clinical characteristics of the participants were collected (age, gender, highest level of education, substance use, and current addiction treatment):

- Measurements in the Addictions for Triage and Evaluation [28] was administered to collect information on substance use and SUDs in the past month and lifetime.
- The 21-item Depression Anxiety Stress Scale (DASS) was used to measure depression, anxiety, and stress [29-31]. The DASS-21 is a self-report questionnaire consisting of 21 items, with 7 items per subscale: depression, anxiety, and stress. Patients were asked to score every item on a scale from 0 (did not apply to me at all) to 3 (applied to me very much). Sum scores were computed by summing the scores on the items per scale and multiplying them by a factor of 2. Sum scores for the total DASS range between 0 and 120, and those for each of the subscales may range between 0 and 42.
- The Manchester Short Assessment of Quality of Life (MANSA) questionnaire was administered to measure quality of life [32]. The questionnaire contained 12 questions about satisfaction with life as a whole, including occupational status (eg, job and sheltered employment), financial situation, number and quality of friendships, leisure activities, accommodation, personal safety, people with whom the patient lives (or living alone), sex life, relationship with family, physical health, and mental health. Satisfaction was rated on a 7-point scale (1=negative extreme; 7=positive extreme).

#### Intervention

**Figure 1:**
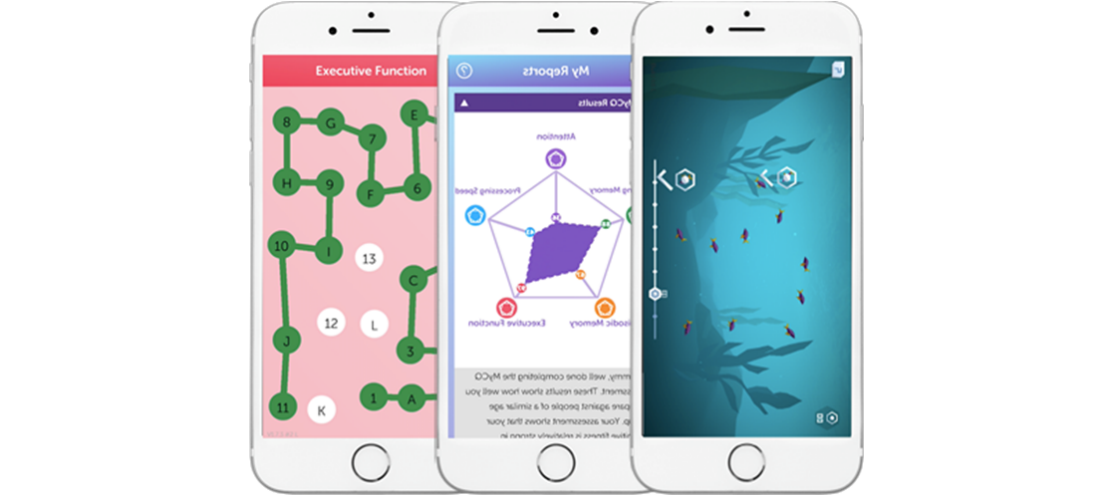
Screenshots of one of the tests and overview page of the MyCognition Quotient neurocognitive assessment, with scores on the five domains assessed (attention, executive functions, episodic memory, and processing speed). On the right, a screenshot of one of the AquaSnap serious games [44].

In this study, 2 smartphone apps developed by MyCognition were used. These apps are available on the web and can be used at home without the help of a trained supervisor. Figure 1 [33] shows a screenshot of these apps.

The app MyCQ assesses cognitive functioning in 5 domains: attention, processing speed, working memory, episodic memory, and executive function. Every subtest starts with a practice trial. Attention is measured using a choice reaction time test, asking the participant to tap the screen when a red dot appears. The processing speed is measured by tapping the screen when a stimulus appears. Working memory is tested using a 2-back task, where participants have to respond when a stimulus matches a picture 2 steps earlier in the sequence. Visual memory is tested by displaying a series of pictures to be remembered. Hereafter, the participant presses a button if they recognize the picture in a new list. Executive functioning is tested with a trail-making task in which the participant has to alternately connect letters with numbers. Latency (reaction time) and accuracy (percentage correct) scores are recorded for each domain.

The second app (AquaSnap) is a videogame that improves cognitive functions and targets the 5 cognitive domains assessed by MyCQ. The training program adapts to the individual MyCQ assessment scores and AquaSnap training performance. The app consists of 7 games that each train one or more cognitive domains. In the games, the player is a submarine that dives underwater and can discover different parts of the ocean. Taking the best pictures of fish and completing missions provides the player experience and currency, which can be used to discover new areas. A detailed description can be found in the study by Domen et al [25].

#### Outcome Measures

##### Feasibility of Engagement

In this study, the apps were considered feasible if 24.8% (57/229) of the recruited patients completed the MyCQ assessment and, of these, 60% (34/57) managed to play AquaSnap for a minimum of 15 minutes [34,35].

##### Acceptability of the Apps

A questionnaire was administered to assess the acceptability of the 2 smartphone apps. Questions about ease of use and likeability were rated on a 5-point Likert scale, where 1=totally disagree, 2=disagree, 3=neither agree nor disagree, 4=agree, and 5=totally agree.

The use of the apps was assumed to be acceptable if at least 30% (14/46 and 10/34, respectively) of the participants who filled out the questionnaire rated the apps positively in terms of ease of use and likeability, that is, if they rated it with a score of 4 (agree) or 5 (totally agree) on a 5-point Likert scale [35].

### Procedure

Participants were instructed to download the apps *MyCQ* and *AquaSnap* from the App or Play Store on their smartphone—with an information leaflet and email sent to them. Logging into the app required a log-in code that was provided by the therapist. If participants had difficulties downloading the app, the therapist helped them with it in a treatment session. All participants were allocated a participant number to be used for the app during its download, and no participant-related identifiers were captured in electronic data files. Electronic data from the smartphone app were synchronized to a password-protected cloud database. The playing time of AquaSnap and the performance of the MyCQ tests were tracked for 6 weeks. In a separate password-protected electronic data file, participant numbers were connected to electronic patient file numbers to be able to contact the participants during the study period. The participants were instructed to start with the MyCQ assessment because the AquaSnap game adapted to the MyCQ test results. Every week, if little or no activity was observed, the participant was called and, if reached, motivated to use the apps. It was advised to play the AquaSnap game for 15 minutes daily, as in previous studies, longer playing times were advised, but shorter times were played [26,36,37]. To stimulate playing the game, the participants who had played AquaSnap for >225 minutes received a €15 (US $15.30) bol.com gift card. To assess acceptability, the completion of at least one MyCQ assessment and a minimum playing time of 15 minutes on AquaSnap was set to ensure that participants had some experience in answering a questionnaire about the use of the apps. In total, 41.8% (46/110) of participants who completed an assessment and 58% (34/59) who played AquaSnap were asked to answer a questionnaire about the use of the apps, which earned them a €10 (US $10.20) gift voucher. The participants were free to stop playing the game at any moment. Because the primary aim was to assess feasibility and acceptability, no additional minimum time, besides the 15-minute limit, was used in the exploratory analyses for the effects of playing AquaSnap on cognition. Assessments of MyCQ, DASS, MANSA, and Measurements in the Addictions for Triage and Evaluation were performed at the start of the treatment and after 6 weeks.

### Analyses

Data were analyzed using SPSS Statistics (version 26; IBM Corp), and the statistical significance was set at .05. The group of participants who performed at least one assessment on their smartphone and the group of participants who did not use the apps at all were compared at baseline based on age, gender, education level, substance use, DASS, MANSA, and treatment type. A *t* test (2-tailed) was performed for continuous variables and a chi-square test for categorical variables (or Mann-Whitney *U* test or Fisher exact test, respectively, as appropriate). Within the group of participants who performed at least one MyCQ assessment, the same comparisons were made between participants who played AquaSnap for at least 15 minutes and those who did not. Mann-Whitney *U* tests were performed to compare the baseline MyCQ scores among the groups. For the MyCQ, we considered both latency (milliseconds) and accuracy (% true) for each of the 5 cognitive domains; therefore, the *P* value was set at .05/2=.03.

The acceptability of the smartphone apps was qualitatively analyzed based on the answers to the questionnaires.

Explorative Spearman correlation analyses were performed between the number of playing minutes on AquaSnap and the change in scores between the first and second MyCQ measurements. For latency, the change scores were calculated by subtracting the latency (speed) in milliseconds at follow-up from the latency in milliseconds at the start. For accuracy, the change scores were calculated by subtracting the accuracy in % true at the start from the % true at the follow-up.

## Results

### Overview

A total of 229 patients (151/229, 65.9% men) seeking addiction treatment for SUD were interested in participating in the study and received a log-in code to use the MyCQ and AquaSnap apps. The mean patient age was 42 (SD 12.7; range 19.4-74.8) years. The patient characteristics are presented in Table 1.

**Table 1:** Patient characteristics in the total sample and groups that used and did not use the app MyCognition Quotient (MyCQ).

Patient characteristics		Total	MyCQ	Non-MyCQ	*P* value^a^	Test
**Gender, n (%)**		229^b^ (100)	110 (100)	119 (100)	.58	Chi-square
	Male	152 (66.4)	75 (68.2)	77 (64.7)		
	Female	77 (33.6)	35 (31.8)	42 (35.3)		
Age (years), mean (SD)		42.0 (12.7)	43.7 (12.9)	40.4 (12.3)	.06	Mann-Whitney
**Level of education,** **n (%)**		174 (100)	87 (100)	87 (100)	*.03* ^c^	Chi-square
	Low	20 (11.5)	5 (5.7)	15 (17.2)		
	Average	63 (36.2)	30 (34.5)	33 (37.9)		
	High	91 (52.3)	52 (59.8)	39 (44.8)		
**Major substance, n (%)**		217 (100)	105 (100)	112 (100)	.25	Chi-square
	Alcohol	101 (46.5)	53 (50.5)	48 (42.8)		
	Cannabis	47 (21.7)	20 (19)	27 (24.1)		
	Cocaine	24 (11.1)	10 (9.5)	14 (12.5)		
	Nicotine	15 (6.9)	8 (7.6)	7 (6.3)		
	Stimulants	9 (4.1)	2 (1.9)	7 (6.3)		
	Sedatives	7 (3.2)	2 (1.9)	5 (4.5)		
	Other	6 (2.8)	5 (4.8)	1 (0.9)		
	Gambling	8 (3.7)	5 (4.8)	3 (2.7)		
**Treatment setting, n (%)**		174 (100)	87 (100)	87 (100)	.45	Chi-square
	Policlinic	96 (55.2)	51 (58.6)	45 (51.7)		
	Daycare	41 (23.6)	17 (19.5)	24 (27.6)		
	Clinic	37 (21.3)	19 (21.8)	18 (20.7)		
**DASS-t0^d^, mean (SD)**		149 (100)	75 (100)	74 (100)		*t* test (2-tailed)
	Depression	15.8 (11.2)	17.4 (11.7)	14.2 (10.4)	.08	
	Anxiety	10.4 (9.1)	10.6 (9.6)	10.2 (8.5)	.82	
	Stress	15.9 (8.8)	16.5 (8.7)	15.2 (8.9)	.35	
	Total	42.0 (25.8)	44.5 (26.9)	39.6 (24.7)	.25	
**MANSA-t0^e^, mean (SD)**		136 (100)	69 (100)	67 (100)		*t* test (2-tailed)
	Total	51.8 (13.3)	51.6 (12.9)	52.1 (13.7)	.85	

^a^Between-group comparisons.

^b^Because of missing data, the n included is mentioned separately.

^c^Significant at *.*05 level

^d^DASS-t0: Depression Anxiety Stress Scale scores at baseline.

^e^MANSA-t0: Manchester Short Assessment of Quality of Life scores at baseline.

### Feasibility of Engagement

Of the 229 recruited patients, 110 completed the MyCQ assessment, providing an acceptance rate of 48%. Of these 110 MyCQ completers, 59 (53.6%) started playing AquaSnap, which was 25.8% (59/229) of the originally recruited patients.

### Comparison of the Participants Who Performed at Least One Assessment With Those Who Did Not Download the Apps

Of the patients who received a log-in code, 48% (110/229) downloaded the MyCQ app and completed at least one assessment. The group of 110 persons who completed the assessment did not differ in age, gender distribution, substance use, treatment setting, DASS, or MANSA scores from the 119 persons who did not download the apps (Table 1). The group that completed the assessment was significantly more educated than the group that did not download the apps (χ^2^_2_=7.3; *P*=.03).

### Comparison Within the MyCQ User Group Between AquaSnap Players and Nonplayers

In total, 59 participants played AquaSnap for a minimum of 15 (mean 194, SD 265, range 15-1032) minutes. They did not differ from the 51 participants who did not play AquaSnap in terms of age, substance use, treatment setting, DASS, or MANSA scores (Table 2). There were significantly more women (25/59, 42%) in the AquaSnap group than in the non-AquaSnap group (10/51, 20%; χ^2^_1_=6.5; *P*=.01). The educational level did not differ significantly between the AquaSnap and non-AquaSnap groups (χ^2^_2_=5.6; *P*=.06). Baseline MyCQ scores did not differ between AquaSnap players and nonplayers (Table 3), except for episodic memory accuracy (*U*=1086; *P*=.01), which was better for AquaSnap players than for nonplayers.

**Table 2:** Patient characteristics of the participants who used the MyCognition Quotient app and per those who used and did not use the AquaSnap app.

Patient characteristics			Total		AquaSnap		Non-AquaSnap		*P* value^a^		Test
**Gender, n (%)**			110^b^ (100)		59 (100)		51 (100)		.01		Chi-square
	Male	75 (68.2)		34 (57.6)		41 (80.4)					
	Female	35 (31.8)		25 (42.4)		10 (19.6)					
Age (years), mean (SD)			43.7 (12.9)		44.8 (12.4)		42.6 (13.5)		.27		Mann-Whitney
**Level of education, n (%)**			87 (100)		46 (100)		41 (100)		.06		Chi-square
	Low	5 (5.7)		4 (8.7)		1 (2.4)					
	Average	30 (34.5)		11 (23.9)		19 (46.3)					
	High	52 (59.8)		31 (67.4)		21 (51.2)					
**Major substance, n (%)**			105 (100)		57 (100)		48 (100)		.29		Chi-square
	Alcohol	53 (50.5)		29 (50.9)		24 (50)					
	Cannabis	20 (19)		12 (21.1)		8 (16.7)					
	Cocaine	10 (9.5)		5 (8.8)		5 (10.4)					
	Nicotine	8 (7.6)		6 (10.5)		2 (4.2)					
	Stimulants	2 (1.9)		2 (3.5)		0 (0)					
	Sedatives	2 (1.9)		0 (0)		2 (4.2)					
	Other	5 (4.8)		2 (3.5)		3 (6.3)					
	Gambling	5 (4.8)		1 (1.8)		4 (8.3)					
**Treatment setting, n (%)**			87 (100)		47 (100)		41 (100)		.40		Chi-square
	Policlinic	51 (58.6)		30 (65.2)		21 (51.2)					
	Daycare	17 (19.5)		8 (17.4)		9 (22)					
	Clinic	19 (21.8)		8 (17.4)		11 (26.8)					
**DASS-t0^c^, mean (SD)**			75 (100)		44 (100)		31 (100)				*t* test, 2-tailed
	Depression	17.4 (11.7)		17.5 (10.8)		17.3 (13.1)		.95			
	Anxiety	10.6 (9.6)		11.6 (10.4)		9.0 (8.3)		.25			
	Stress	16.5 (8.7)		16.9 (8.8)		16.1 (8.6)		.70			
	Total	44.5 (26.9)		46 (27.1)		42.4 (26.7)		.58			
**MANSA-t0^d^, mean (SD)**			69 (100)		40 (100)		29 (100)				*t* test, 2-tailed
	Total	51.6 (12.9)		50.4 (12.6)		53.3 (13.5)		.35			

^a^Between-group comparisons.

^b^Because of missing data, the n included is mentioned separately.

^c^DASS-t0: Depression Anxiety Stress Scale scores at baseline.

^d^MANSA-t0: Manchester Short Assessment of Quality of Life scores at baseline.

**Table 3:** Mann-Whitney U test results for the comparison of baseline MyCognition Quotient (MyCQ) scores between participants who used and did not use AquaSnap.

MyCQ assessment	AquaSnap (n^a^=59), mean (SD)	Non-AquaSnap (n=50), mean (SD)	*P* value
Attention latency^b^	516 (106)	515 (139)	.89
Attention accuracy^c^	95 (10)	93 (17)	.69
Processing speed latency	362 (70)	356 (75)	.94
Processing speed accuracy	96 (5)	93 (17)	.22
Working memory latency	1312 (403)	1283 (444)	.58
Working memory accuracy	89 (14)	87 (15)	.41
Episodic memory latency	1128 (205)	1202 (428)	.56
Episodic memory accuracy	92 (7)	89 (8)	*.01* ^d^
Executive functioning latency	1417 (1298)	1716 (1535)	.16
Executive functioning accuracy	91 (14)	89 (16)	.21

^a^Owing to missing data for the subtest attention, n was 54 and 47, respectively.

^b^Latency in milliseconds.

^c^Accuracy in % true.

^d^This correlation was significant at the .03 level.

### Acceptability of the Apps

Of the 110 participants who performed the MyCQ assessment, 46 (41.8%) were asked to complete a questionnaire on the acceptability of the MyCQ assessment (Table 4). Of the 46 participants who completed the assessment, 38 (83%) enjoyed taking it, whereas only 3 (7%) disliked it. The majority (35/46, 76%) rated the app as easy. Furthermore, 74% (34/46) believed that the app provided insight into their brain functions. Half (23/46, 50%) of the participants who completed the questionnaire did not believe that the MyCQ assessments contributed to their addiction treatment and did not continue taking the measurements after finishing their treatment.

**Table 4:** Frequencies (%) perception questionnaire regarding serious gaming apps.

		Ratings^a^, n (%)						Value, mean (SD)
		1	2	3	4	5		
**MyCQ^b^ assessment (n=46)**								
	1. Did you like the MyCQ task?	1 (2)	2 (4)	5 (11)	28 (61)	10 (22)	4 (0.8)	
	2. Did the MyCQ task contribute to your addiction treatment?	13 (28)	10 (22)	14 (30)	7 (15)	2 (4)	2.5 (1.2)	
	3. Did the MyCQ task provide insight into your brain functions?	4 (9)	3 (7)	5 (11)	31 (67)	3 (7)	3.6 (1)	
	4. Would you continue with MyCQ after your treatment?	15 (33)	10 (22)	5 (11)	13 (28)	3 (7)	2.5 (1.4)	
	5. Was MyCQ easy in use?	1 (2)	6 (13)	4 (9)	20 (43)	15 (33)	3.9 (1)	
**AquaSnap (n=34)**								
	1. Did you like the AquaSnap game?	1 (3)	11 (32)	8 (24)	8 (24)	6(18)	3 (1)	
	2. Do you think your brain functions are improved by playing AquaSnap?	4 (12)	7 (21)	7 (21)	14 (41)	2 (6)	2.8 (1.3)	
	3. Did AquaSnap contribute to your addiction treatment?	9 (27)	5 (15)	8 (24)	10 (29)	2 (6)	2.5 (1.3)	
	4. Did playing AquaSnap help you better manage your addiction?	11 (32)	11 (32)	7 (21)	4 (12)	1 (3)	2.1 (1.1)	
	5. Do you think the chance to relapse have diminished through AquaSnap?	13 (38)	7(21)	10 (29)	3 (9)	1 (3)	2.1 (1.1)	
	6. Would you continue with AquaSnap after your treatment?	11 (32)	5 (15)	8 (24)	7 (21)	3 (9)	2.4 (1.4)	
	7. Did you find AquaSnap easy in use?	1 (3)	6 (18)	4 (12)	17 (50)	6 (18)	3.3 (1.3)	

^a^1=totally disagree, 2=disagree, 3=neither agree nor disagree, 4=agree, and 5=totally agree.

^b^MyCQ: MyCognition Quotient.

Of the 59 participants who played AquaSnap for at least 15 minutes, 34 (58%) completed a questionnaire on the acceptability of the AquaSnap app. Almost half (16/34, 47%) of the participants believed that playing the game improved their brain functions. The majority (23/34, 68%) rated the app as easy. Furthermore, 41% (14/34) of the participants enjoyed playing the game, whereas 35% (12/34) did not like it. Moreover, 35% (12/34) of the participants who completed the questionnaire believed that playing AquaSnap contributed to their addiction treatment, and 30% (10/34) stated that they would continue playing the game after finishing the treatment. The app’s ratings positively correlated with the number of minutes played (Table 5).

**Table 5:** Spearman ρ correlations between the number of playing minutes with AquaSnap and AquaSnap ratings (n=34).

AquaSnap questions^a^	1	2	3	4	5	6	7
ρ	0.8	0.5	0.5	0.5	0.5	0.5	−0.02
*P* value (2-tailed)	<.001	.002	.001	.008	.005	.004	.93

^a^See Table 4.

### Explorative Analyses Effectiveness AquaSnap

The MyCQ assessment scores and AquaSnap playing time were inspected for outliers by using box plots. In total, 4 MyCQ data points were missing for the cognitive domain *attention*, and 2 were missing for *processing speed*, most likely because of technical failure.

The number of minutes played with AquaSnap correlated with the change in working memory latency time between the first and second MyCQ assessments; more playing minutes were associated with a decrease in working memory reaction time (Spearman ρ=0.4; *P*=.01). An increase in AquaSnap playing minutes was associated with a decrease in executive functioning accuracy between the first and second MyCQ assessments (Spearman ρ=−0.3; *P*=.02). No other significant correlations were observed between the first and second assessments (Table 6).

**Table 6:** Spearman ρ correlations between the number of playing minutes with AquaSnap and change scores^a^ between the first (T1) and second (T2) MyCognition Quotient (MyCQ) assessments.

		Attention latency		Attention accuracy	Processing speed latency			Processing speed accuracy	Working memory latency		Working memory accuracy		Episodic memory latency		Episodic memory accuracy		Executive functioning latency		Executive functioning accuracy	
n	42		42			44	44			46		46		46		46		46		46
ρ	0.1		0.1			0.2	−0.1			*0.4* ^b^		0.0		0.0		0.2		−0.2		−*0.3*^b^
*P* value	.59		.66			.25	.72			*.01* ^b^		.88		.84		.22		.14		*.02* ^b^

^a^Latency (speed in milliseconds) change scores T1 minus T2; accuracy (% true) change scores T2 minus T1.

^b^Correlation is significant at the .03 level (2-tailed).

## Discussion

### Principal Findings

This study investigated the feasibility and acceptability of 2 serious gaming smartphone apps in a group of patients with a SUD in an addiction treatment setting. Both the MyCQ assessment and AquaSnap cognitive training apps were offered in addition to regular addiction care. This study showed that the use of MyCQ is feasible among patients with SUDs who are interested and highly educated. Approximately half of the interested patients actually used MyCQ. However, the feasibility of using the AquaSnap training app was lower with only 25.8% (59/229) of the initially interested patients. This percentage is lower than that in a recent study of patients with SUD on the feasibility of a smartphone attention bias app. Zhang et al [35] investigated the use of an attention bias modification app on a smartphone in 40 inpatients with SUD (predominantly opioid use disorder) and found 75% acceptance and 63% adherence rates. It is important to note that the study by Zhang et al [35] was conducted in a group of inpatients admitted for rehabilitation and lasted only a week. This is in contrast to this study, in which both inpatients and outpatients were included, who were followed up for 6 weeks. Moreover, participation in this study was entirely voluntary, and participants could stop at any time; therefore, adherence is expected to be higher when implemented as a regular part of treatment.

Highly educated patients were more likely to start using MyCQ. This finding can be partly explained by the fact that highly educated patients are more familiar with scientific research [38]. In addition, highly educated people have better executive functions [39], suggesting that they are better at organizing, staying focused, and exerting self-control [40]. These characteristics promote participation in voluntary studies such as this one. More women than men played the AquaSnap training app. This may indicate volunteer bias [41], although we found no such bias in initial participation in the MyCQ assessment.

Regarding acceptability, our study showed mixed results. The MyCQ assessment can be defined as acceptable for the group that was presented with the questionnaire, with 76% (35/46) and 83% (38/46) of the participants reporting it to be easy and likable, respectively. Although the AquaSnap game was rated as easy by 68% (23/34) of the participants, only 41% (14/34) enjoyed playing the game. A large majority (34/46, 74%) believed that MyCQ provided insight into their brain functions, whereas only half (16/34, 47%) of the participants believed that playing AquaSnap improved their brain functions. Only 15% (5/34) believed that playing the AquaSnap helped them manage their addiction, which is much lower than the 36% found in the study by Zhang et al [35]. One reason may be that the game used in this study (clicking photographs of fishes) was perceived as less relevant to the addiction problem than the game used in a study by Zhang et al [35] (pushing away substance-related pictures).

The AquaSnap app rating was positively correlated with the number of minutes played (Spearman ρ ranging from 0.5 to 0.7; *P*<.05). It is possible that the participants played more because they liked the game better, but it could also be that they liked the game better because they played more.

Explorative analysis showed that more playing minutes on AquaSnap was related to shorter reaction times in the domain of working memory but more errors in the domain of executive functioning, as measured by the MyCQ assessment. These associations could be due to factors other than playing the game, such as changes in substance use or the length of substance abstinence. Moreover, the divergent validity of working memory and executive functioning tasks in MyCQ was found to be limited [25]. Therefore, these results should be interpreted with caution.

### Strengths and Limitations

A strength of this study is that it had a naturalistic design and was conducted within regular addiction care, with the feasibility of using a smartphone app investigated in both outpatient and inpatient addiction settings. Patients can play serious games in their own environments at times of their choice. Thus, the ecological validity of this study was high. Participation was low threshold, it took little effort to use the apps, and there were no use restrictions, which increased the number of participants and thus the reliability of the data. Furthermore, this study evaluated the opinions of patients on 2 different smartphone apps, both pertaining to cognitive assessment and cognitive training, providing relevant information for clinical practice.

This study has some limitations. The disadvantage of using a naturalistic design is that there is no standardized implementation of the apps. The circumstances under which the apps were used may have differed, because participants could decide when and where they wanted to use the apps. Furthermore, in addition to the advantages of accessibility, the use of personal phones has several potential complications, such as differences in screen size and processing capacity and speed.

In this study, we defined the recruited population as patients interested in participating. However, we do not know exactly how much of the total population was informed about the study. We know that 41.9% (78/186) of the informed inpatients have participated, but data on this are lacking for the outpatient population. Nevertheless, the participants were comparable with the total population in this department in the year of recruitment in terms of age, primary SUD, gender, mood, and quality of life. In addition, there was high variability between the participants in terms of the time they spent playing AquaSnap. This hampered the interpretation of the results. Moreover, because participation was voluntary and not an integral part of usual care, adherence rates were low. It is already difficult to monitor addictive patients, because the overall no-shows and dropout rates are known to be high [7]. Another potential confounding factor that we did not measure was a potential difference in familiarity with mobile technology [42]. This may have induced a higher selection of participants with better technological skills, which may have resulted in a more positive evaluation of the apps due to the overinclusion of participants with higher technology readiness.

### Recommendations for Future Research

Future research should include and evaluate the use of smartphone apps as an integral part of usual care. Thus, adherence can be monitored more closely, and there is a greater chance that more people with a lower education level will participate. In addition, patients should be encouraged to train more intensively. More intensive cognitive training has shown positive effects on working memory and alcohol consumption [43,44]. Although the acceptability of the MyCQ assessment was good, the acceptability of the training app AquaSnap was lower. For future research, this app should be made more attractive, or an alternative serious game should be chosen that is also more in line with the addiction problem in terms of content. One of our findings was that the group of patients who used the apps had a higher education level than the group that did not start using the apps. Thus, for feasibility in future studies, attention needs to be paid to engaging patients with different educational levels. Future research using, for example, focus groups could investigate the needs and interests of patients with SUD from different educational levels for these cognitive training interventions.

In addition, it is recommended that initial work be conducted to understand the characteristics of the specific target population and their ownership of smartphone devices [42] before implementation. In clinical practice, the use of the MyCQ app has potential, given the high prevalence of cognitive impairment in this group and the advice to screen for cognitive impairment early in treatment [11]. The fact that app assessment takes an assessor less time than the classic paper-and-pencil test may result in lower costs for cognitive assessment.

### Conclusions

In conclusion, our study shows that the use of a smartphone app for cognitive assessment in patients with SUDs who are interested and highly educated is feasible, and for the subgroup who filled out the questionnaire, it was acceptable. However, our data also highlight that the use of a smartphone app for cognitive training via serious gaming is less feasible in this group of patients. Improvement of the app and motivation of clients to increase the use of serious games is needed. Despite these limitations, the present results provide support for future research investigating the use of smartphone apps for cognitive assessment and cognitive training in relation to the treatment of SUD because participation and acceptability rates were sufficient.

## Abbreviations

DASS: Depression Anxiety Stress Scale
MANSA: Manchester Short Assessment of Quality of Life
MyCQ: MyCognition Quotient
SUD: substance use disorder
